# Efficacy of a Community-Based Technology-Enabled Physical Activity Counseling Program for People With Knee Osteoarthritis: Proof-of-Concept Study

**DOI:** 10.2196/jmir.8514

**Published:** 2018-04-30

**Authors:** Linda C Li, Eric C Sayre, Hui Xie, Ryan S Falck, John R Best, Teresa Liu-Ambrose, Navi Grewal, Alison M Hoens, Greg Noonan, Lynne M Feehan

**Affiliations:** ^1^ Department of Physical Therapy University of British Columbia Vancouver, BC Canada; ^2^ Arthritis Research Canada Richmond, BC Canada; ^3^ Faculty of Health Sciences Simon Fraser University Burnaby, BC Canada; ^4^ Mary Pack Arthritis Program Vancouver General Hospital Vancouver, BC Canada

**Keywords:** osteoarthritis, physical activity, wearables, goal setting, physiotherapy, eHealth

## Abstract

**Background:**

Current practice guidelines emphasize the use of physical activity as the first-line treatment of knee osteoarthritis; however, up to 90% of people with osteoarthritis are inactive.

**Objective:**

We aimed to assess the efficacy of a technology-enabled counseling intervention for improving physical activity in people with either a physician-confirmed diagnosis of knee osteoarthritis or having passed two validated criteria for early osteoarthritis.

**Methods:**

We conducted a proof-of-concept randomized controlled trial. The immediate group received a brief education session by a physical therapist, a Fitbit Flex, and four biweekly phone calls for activity counseling. The delayed group received the same intervention 2 months later. Participants were assessed at baseline (T0) and at the end of 2 months (T1), 4 months (T2), and 6 months (T3). Outcomes included (1) mean time on moderate-to-vigorous physical activity (MVPA ≥3 metabolic equivalents [METs], primary outcome), (2) mean time on MVPA ≥4 METs, (3) mean daily steps, (4) mean time on sedentary activities, (5) Knee Injury and Osteoarthritis Outcome Score (KOOS), and (6) Partners in Health scale. Mixed-effects repeated measures analysis of variance was used to assess five planned contrasts of changes in outcome measures over measurement periods. The five contrasts were (1) immediate T1-T0 vs delayed T1-T0, (2) delayed T2-T1 vs delayed T1-T0, (3) mean of contrast 1 and contrast 2, (4) immediate T1-T0 vs delayed T2-T1, and (5) mean of immediate T2-T1 and delayed T3-T2. The first three contrasts estimate the between-group effects. The latter two contrasts estimate the effect of the 2-month intervention delay on outcomes.

**Results:**

We recruited 61 participants (immediate: n=30; delayed: n=31). Both groups were similar in age (immediate: mean 61.3, SD 9.4 years; delayed: mean 62.1, SD 8.5 years) and body mass index (immediate: mean 29.2, SD 5.5 kg/m^2^; delayed: mean 29.2, SD 4.8 kg/m^2^). Contrast analyses revealed significant between-group effects in MVPA ≥3 METs (contrast 1 coefficient: 26.6, 95% CI 4.0-49.1, *P*=.02; contrast 3 coefficient: 26.0, 95% CI 3.1-49.0, *P*=.03), daily steps (contrast 1 coefficient: 1699.2, 95% CI 349.0-3049.4, *P*=.02; contrast 2 coefficient: 1601.8, 95% CI 38.7-3164.9, *P*=.045; contrast 3 coefficient: 1650.5, 95% CI 332.3-2968.7; *P*=.02), KOOS activity of daily living subscale (contrast 1 coefficient: 6.9, 95% CI 0.1-13.7, *P*=.047; contrast 3 coefficient: 7.2, 95% CI 0.8-13.6, *P*=.03), and KOOS quality of life subscale (contrast 1 coefficient: 7.4, 95% CI 0.0-14.7, *P*=.049; contrast 3 coefficient: 7.3, 95% CI 0.1-14.6, *P*=.048). We found no significant effect in any outcome measures due to the 2-month delay of the intervention.

**Conclusions:**

Our counseling program improved MVPA ≥3 METs, daily steps, activity of daily living, and quality of life in people with knee osteoarthritis. These findings are important because an active lifestyle is an important component of successful self-management.

**Trial Registration:**

ClinicalTrials.gov NCT02315664; https://clinicaltrials.gov/ct2/show/NCT02315664 (Archived by WebCite at http://www.webcitation.org/6ynSgUyUC)

## Introduction

Arthritis is the most common cause of severe chronic pain and disability worldwide. Analysis by the Arthritis Alliance of Canada estimates one new diagnosis of osteoarthritis (OA) every 60 seconds, resulting in nearly 30% of the employed labor force having difficulties working due to OA [1]. Current evidence supports the use of physical activity to manage OA due to its beneficial effects on pain, mobility, and quality of life [2,3]. It has been shown that moderate weight-bearing activities improve joint health by preserving glycosaminoglycan content in cartilage [4,5]. Furthermore, specific training that involves functional activities improves balance and proprioception, which in turn can contribute to improving pain and mobility [6,7]. The OA Research Society International recommends the use of physical activity and therapeutic exercise as a first-line treatment of knee OA [8]. Public health guidelines recommend more than 150 minutes a week of moderate-to-vigorous physical activity (MVPA) performed in bouts of 10 minutes or more [9]; however, a 2013 systematic review reported that only 13% of people with OA met this recommendation [10]. This concurs with another study using accelerometers that more than 90% of people with knee OA did not meet the physical activity guidelines [11]. The findings are particularly alarming because the evidence on the first-line treatment for OA has been consistent over a decade [12]. This represents a major knowledge-to-action gap.

Several modifiable risk factors are associated with low physical activity participation in people with arthritis. These include lack of motivation [13], doubts about the effectiveness of exercise [14], and lack of health professional advice [15]. Once patients start being active, they need feedback on their progress. A Cochrane review reported that “graded exercise activity,” which initially focuses on simple activities and then gradually increases to more challenging ones, is effective for improving adherence in people with chronic musculoskeletal conditions [16]. Progression of activities and goals can be guided by a physical therapist (PT) [16].

We recently demonstrated feasibility of a physical activity counseling program with the use of a Fitbit wrist band in 34 people with knee OA [17]. Compared to controls, those who received the program showed a trend of increased MVPA and perceived self-management capacity after 1 month [17]. The findings supported further research on this program. The purpose of the current study was to assess the efficacy of the program for improving physical activity participation, disease status, and perceived self-management capacity in people with knee OA.

## Methods

### Study Design and Participant Eligibility

Monitor-OA was a proof-of-concept study that used a randomized, delayed-control design, whereby the randomization determined the timing of when the intervention was provided (ie, immediately vs a 2-month delay). As such, efficacy was assessed within a conventional randomized controlled trial (RCT), with an intervention group and a control group, at 2 months, whereas all participants received the intervention beyond this time. This study design is best suited for interventions that include components that are likely beneficial and low risk to participants, such as physical activity counseling.

Eligible individuals were those who had a physician-confirmed diagnosis of knee OA or passed two criteria for early OA: (1) aged 50 years and (2) had experienced pain or discomfort in or around the knee during the previous year lasting more than 28 separate or consecutive days. In a community-based study [18], 191 of 195 (97%) urban-dwelling participants who met these criteria also met the American College of Rheumatology clinical criteria for knee OA [19].

We excluded individuals who:

1. had a diagnosis of inflammatory arthritis, connective tissue diseases, fibromyalgia, or gout;
2. had used disease-modifying antirheumatic drugs or gout medications;
3. had knee arthroplasty;
4. were on a waitlist to receive knee or hip arthroplasty;
5. had any surgery in the back, hip, knee, foot, or ankle joint in the past 12 months;
6. had acute knee injury in the past 6 months;
7. had received a steroid injection or hyaluronate injection in a knee in the last 6 months;
8. had a body mass index (BMI) of 40 kg/m^2^ or higher;
9. did not have an email address or daily access to a personal computer with Internet access;
10. were unable to attend the required education session in person;
11. were using medications that impaired activity tolerance (eg, beta-blockers); and
12. had an inappropriate level of risk for increasing their unsupervised physical activity.

Potential participants completed the Physical Activity Readiness Questionnaire (PAR-Q [20]; 2014 version). If a potential risk was identified by the PAR-Q, physician confirmation in writing was required to ensure that the person was able to be physically active without supervision of a health professional.

Participants were recruited from the Mary Pack Arthritis Program in Vancouver, BC, Canada. Study information was also posted on social media (Facebook, Twitter, Kijiji, and Craigslist) and the Arthritis Research Canada website. In addition, emails about the study were sent by the Arthritis Consumer Experts, a nonprofit patient education organization, to their members. After completing the baseline assessment, eligible participants were randomly assigned to the immediate group or the delayed group (ie, control) in 1:1 allocation ratio. The delayed group received the same intervention as the immediate group after a 2-month wait. We performed randomization using computer-generated random numbers in variable block sizes.

### Intervention

The intervention involved participants attending a 1.5-hour session, where they received (1) 15-minute standardized education about physical activity, (2) a Fitbit Flex, and (3) individual counseling with a study PT who was trained in motivational interviewing [21]. The choice of a face-to-face session over the use of videoconferencing technology was to maximize the opportunity for participants and PTs to established rapport [22,23]. The education portion, delivered in groups of two to four participants, addressed the benefits of an active lifestyle, the detrimental effect of sedentary behavior, and ways to be active without aggravating OA symptoms. The individual counseling portion followed the Brief Action Planning approach [24], whereby PTs guided participants to identify activity goals, develop an action plan, and identify barriers and solutions. The PTs used the SMART (specific, measurable, attainable, relevant, time-bound) principle during goal setting (eg, 30 minutes of brisk walking in the neighborhood in the evening three times a week). Participants were then asked to rate their confidence in executing the plan on a zero to 10 scale, with 10 meaning very confident. The process was repeated until the confidence rating reached 7 or higher out of 10. For sedentary behaviors, the PTs began by asking participants to estimate their sitting time in a normal day and identify ways to break up the sitting time. They then repeated the goal setting and confidence assessment.

Participants then received a Fitbit Flex to be worn at the wrist of the nondominant side 24 hours a day except during water-based activity or when charging. The physical activity data were wirelessly synchronized with Fitbit’s online Dashboard that could be viewed only by the participants and their study PTs. During the intervention period, the PT reviewed the participant’s physical activity on the Dashboard and progressively modified their SMART goals during four biweekly 20-minute phone calls. Participants could also contact the PT via email in-between the scheduled calls. At the end of the intervention, participants could keep the Fitbit.

Four PTs with a primary caseload consisting of patients with arthritis were trained to deliver the education and counseling components. Three of them were from the public sector (Vancouver Coastal Health Authority, Vancouver, BC, Canada), and one worked in a mix of public and private practices. One PT also participated in our previous feasibility study [17] and provided feedback to refine the intervention for the current project. The PTs attended a 2-day introductory motivational interview course offered by the University of British Columbia Extended Learning program. Before data collection, we held two orientation sessions (2 hours each) for the PTs to review the study protocol and practice the counseling component.

### Outcome Measures

Participants were assessed at baseline (T0) and the end of 2 months (T1), 4 months (T2), and 6 months (T3). Our primary outcome measure was mean daily time performing MVPA at ≥3 metabolic equivalents, or METs (MVPA ≥3 METs; performed in both ambulatory and nonambulatory activities throughout the day) measured with SenseWear Mini, a multisensor monitor that was worn on the upper arm over the triceps. SenseWear integrates triaxial accelerometer data, physiological sensor data, and personal demographic information to provide estimates of steps and energy expenditure. Tierney et al [25] showed that SenseWear was a valid tool for estimating energy expenditure during activities of daily living in people with arthritis (intraclass correlation coefficient [ICC]=0.72). A strong relationship was also found between SenseWear and indirect calorimetry measures of energy expenditure for activities of daily living (Pearson *r*=.85) [25]. SenseWear can be worn 24 hours a day; hence, it can capture a full picture of physical activity and the off-body time throughout the day [26,27]. Participants wore a SenseWear for 7 days at each assessment. Almeida et al [28] determined that a minimum of 4 days of wear was required to reliably assess energy expenditure from different levels of physical activity in people with arthritis (ICC >0.80).

We performed additional analysis with a MVPA cut-off at ≥4 METs, which reflected an activity level of brisk walking and higher (ie, purposeful ambulatory activities) [29]. Other secondary outcomes included the daily mean time spent in sedentary activities [30], the Knee Injury and OA Outcome Score (KOOS) [31,32], and the Partners in Health scale [33]. An important feature of SenseWear is its ability to differentiate between sedentary and light activities [34], making it an ideal instrument to assess sedentary activities. For sedentary activities, we calculated the mean daily time spent with an energy expenditure of ≤1.5 METs, occurring in bouts of 20 minutes or more during waking hours [35-38].

The KOOS consists of five subscales: knee pain, stiffness, activity of daily living, sports/recreation, and quality of life. It was originally developed for people recovering from anterior cruciate ligament and meniscus injury and has been validated in people with OA [31,32]. The Partners in Health scale is a 12-item measure designed to assess perceived self-management capacity via subjective knowledge of the health condition and treatment, and perceived self-management behaviors (eg, adopting a healthy lifestyle; Cronbach alpha=.82) [33]. We also tracked self-reported adverse events (falls, cardiovascular and musculoskeletal events) [39] using a monthly log.

### Sample Size Justification and Data Analysis

Our recruitment strategy enabled the study to enroll at least 60 eligible participants over 12 months. For a proof-of-concept study, it is reasonable to expect a moderately large difference between groups after the intervention. Based on our feasibility study, we estimated the standard deviation of the change in MVPA ≥3 METs (primary outcome measure) from T0-T1 to be 40 minutes. This resulted in 81.5% power to detect a 30-minute difference between groups in the T1-T0 change via a two-sided test at alpha level of .05.

Descriptive analysis was used to summarize participant characteristics, comorbid conditions, and adverse events. We generated plots that included means and standard errors for the outcome measures at each time point for both groups for all outcome measures.

An intention-to-treat analysis was performed by a biostatistician who was blinded to the group assignment. Quantile-quantile plots were used to assess normality of the outcome variables. Mixed-effects repeated measures analysis of variance was used to assess five planned contrasts of changes in outcome measures over measurement periods. They were:

1. Contrast 1: immediate group T1-T0 vs delayed group T1-T0;
2. Contrast 2: delayed group T2-T1 vs delayed group T1-T0;
3. Contrast 3: mean of contrast 1 and contrast 2;
4. Contrast 4: immediate group T1-T0 vs delayed group T2-T1; and
5. Contrast 5: mean of immediate group T2-T1 and delayed group T3-T2.

The first three contrasts estimate the effect of the intervention versus control. The latter two contrasts estimate the effect of the delay on the intervention. Contrast 1 is the between-group contrast estimate for the 2-month effect of the program, similar to analysis from a parallel design. Contrast 2 is the within-group contrast estimate for the 2-month effect of the program. One benefit of the delayed-control design is that experimental units can serve as their own control to improve efficiency and precision of treatment effect estimation. To further improve the treatment effect estimation efficiency, we combined contrast 1 and 2 by taking their mean (contrast 3). Contrast 4 examines the 2-month effect of the program when it was delayed for 2 months. Contrast 5 combines data from both groups to assess the lastingness of the effect, when the program ended 2 months before assessment. No adjustment was made for multiple comparisons because type II error is a greater concern than type I error in proof-of-concept studies [40,41].

### Ethics Approval

The research protocol was approved by the University of British Columbia Behavioral Research Ethics Board (application number: H14-01762) and was published in ClinicalTrials.gov (NCT02315664).

## Results

**Figure 1:**
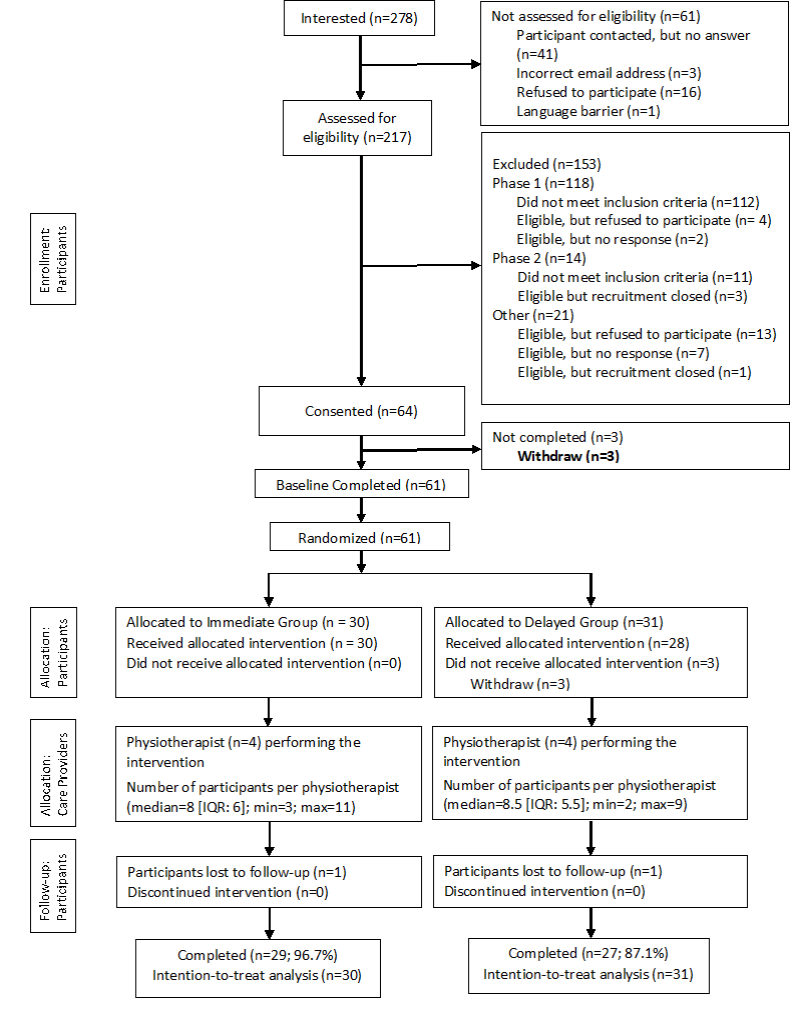
Consolidated Standards of Reporting Trials (CONSORT) flowchart.

In 2015-2016, 278 people indicated an interest to participate and 64 met the eligibility criteria (Figure 1). Of those, we recruited 61 participants (immediate group: n=30, 73%, 22/30 women; delayed group: n=31, 90%, 28/31 women). Both groups were similar in age (immediate group: mean 61.3, SD 9.4 years; delayed group: mean 62.1, SD 8.5 years) and BMI (immediate group: mean 29.2, SD 5.5 kg/m^2^; delayed group: mean 29.2, SD 4.8 kg/m^2^; Table 1).

**Figure 2:**
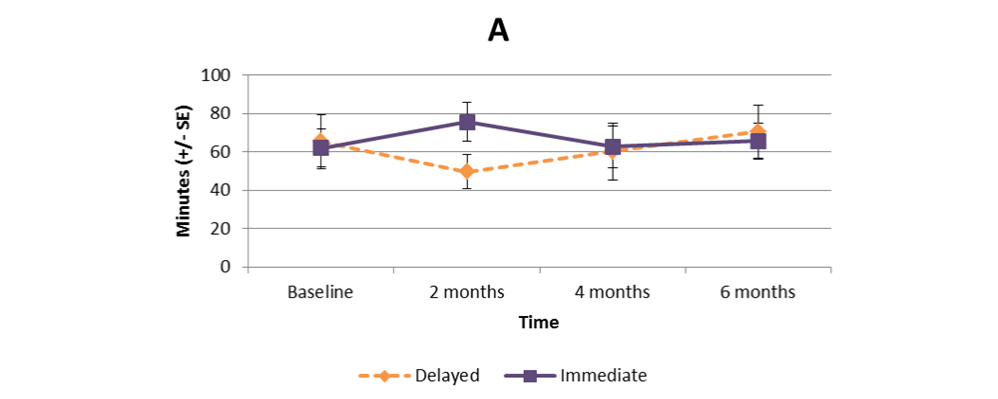
Bouted moderate to vigorous physical activity (≥3 metabolic equivalent tasks [METs]).

**Figure 3:**
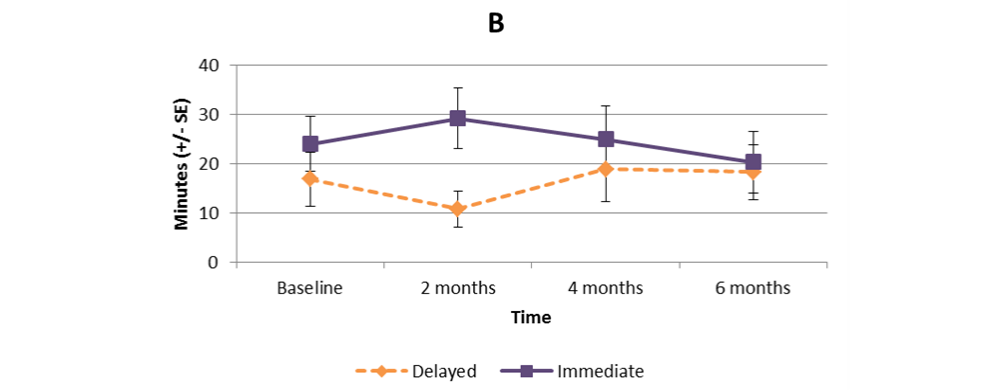
Bouted moderate to vigorous physical activity (≥4 METs).

**Figure 4:**
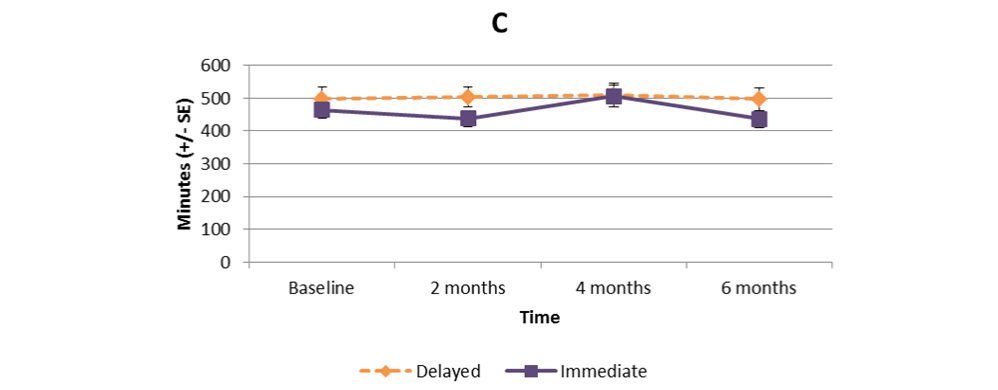
Bouted sedentary time.

**Figure 5:**
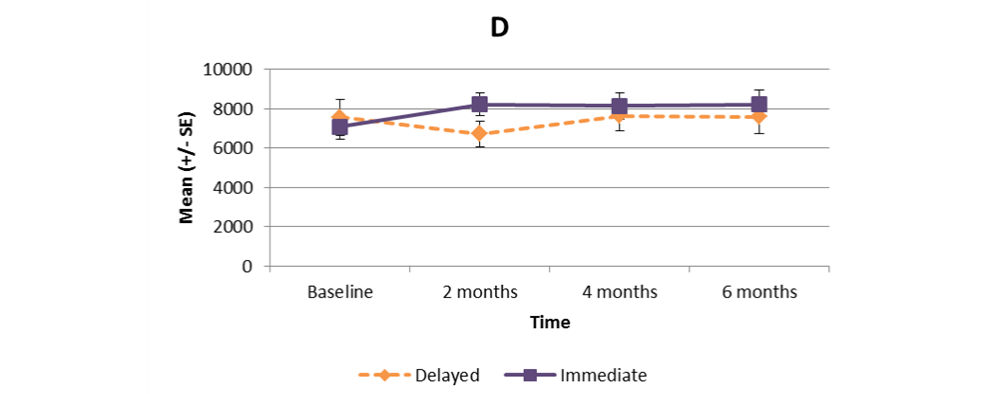
Mean daily step count.

**Figure 6:**
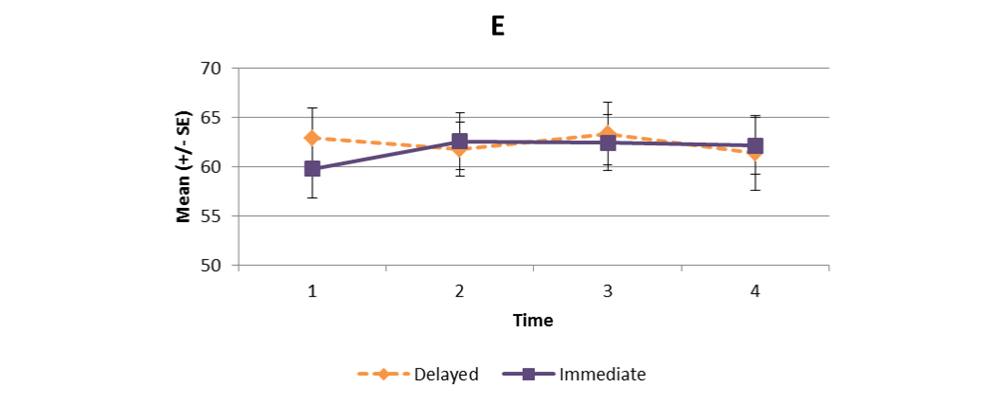
Knee Injury and Osteoarthritis Outcome Score (KOOS) symptoms subscale.

**Figure 7:**
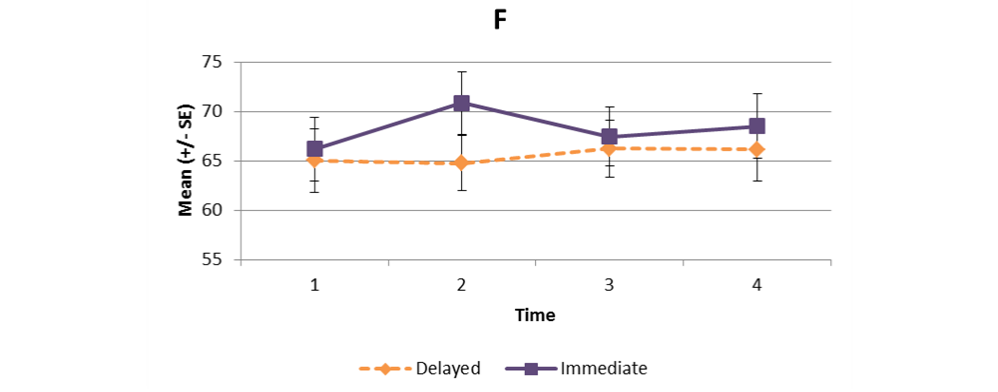
KOOS pain subscale.

**Figure 8:**
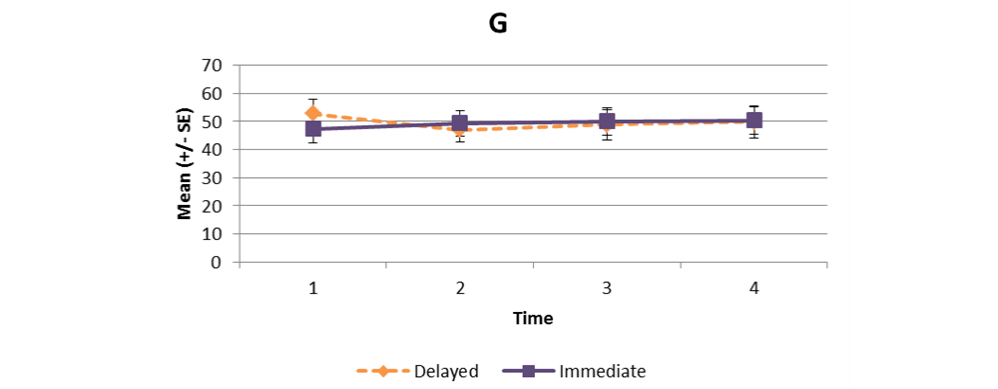
KOOS sports and recreation subscale.

**Figure 9:**
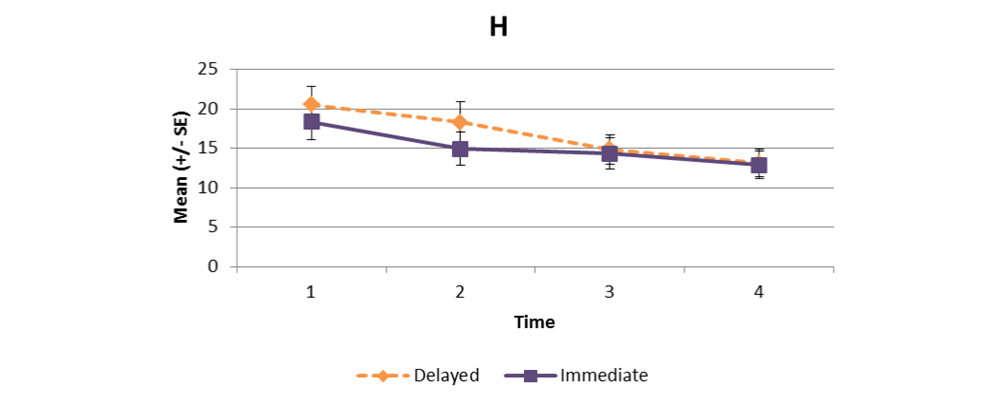
Partners in Health scale.

**Figure 10:**
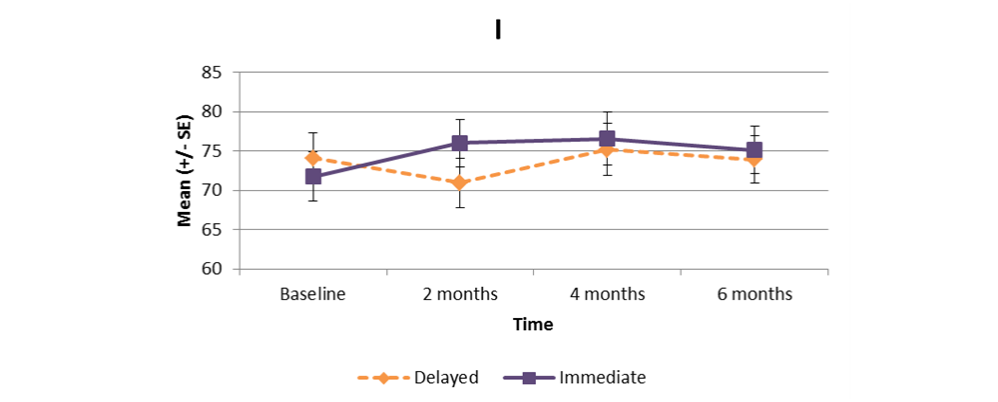
KOOS activities of daily living subscale.

**Figure 11:**
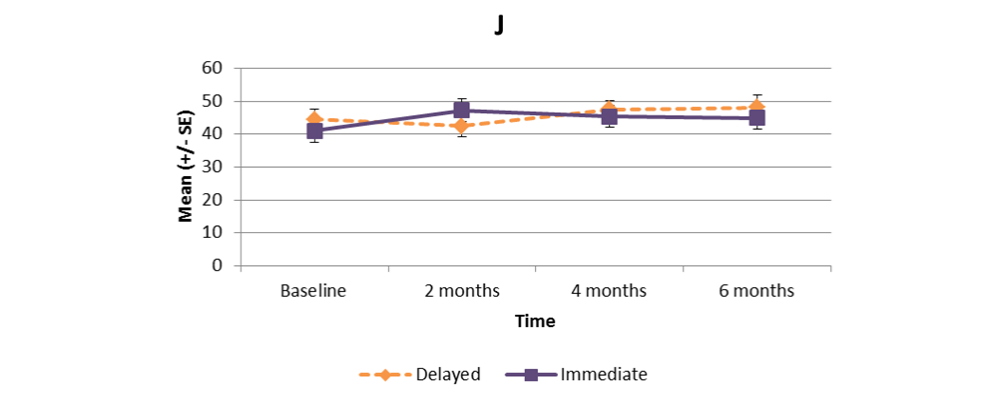
KOOS quality of life subscale.

Multimedia Appendix 1 and Figures 2-11 present the results of outcome measures from four time points. Prespecified contrast analyses revealed significant effects as follows: mean time on MVPA ≥3 METs (contrast 1 coefficient: 26.6, 95% CI 4.0-49.1, *P*=.02; contrast 3 coefficient: 26.0, 95% CI 3.1-49.0, *P*=.03), mean daily steps (contrast 1 coefficient: 1699.2, 95% CI 349.0-3049.4, *P*=.02; contrast 2 coefficient: 1601.8, 95% CI 38.7-3164.9, *P*=.045; contrast 3 coefficient: 1650.5, 95% CI 332.3-2968.7, *P*=.02), KOOS activity of daily living subscale (contrast 1 coefficient: 6.9, 95% CI 0.1-13.7, *P*=.047; contrast 3 coefficient: 7.2, 95% CI 0.8-13.6, *P*=.03), KOOS quality of life subscale (contrast 1 coefficient: 7.4, 95% CI 0.0-14.7, *P*=.049; contrast 3 coefficient: 7.3, 95% CI 0.1-14.6, *P*=.048). We found no significant effect in any outcome measures in the other contrast analyses. No adverse event associated with the intervention (eg, falls, cardiovascular and musculoskeletal events) was reported by participants during the study.

**Table 1:** Baseline characteristics of immediate group and delayed group participants.

Characteristics		All (N=61)	Immediate group (n=30)	Delayed group (n=31)	*P* value^a^
Gender (female), n (%)		50 (82)	22 (73)	28 (90)	.11
Age (years), mean (SD)		61.7 (8.9)	61.3 (9.4)	62.1 (8.5)	.72
**Marital status, n (%)**					.27
	Married/Common law	33 (54)	19 (63)	14 (45)	
	Separated/Divorced	15 (25)	7 (23)	8 (26)	
	Widowed / Never married / Other	13 (21)	4 (13)	9 (29)	
University degree, n (%)		30 (49)	14 (47)	16 (52)	.80
**Gross annual household income (CAN$), n (%)**					.62
	≤12,000	0	0	0	
	12,001-24,000	3 (5)	1 (3)	2 (7)	
	24,001-40,000	6 (10)	4 (13)	2 (7)	
	40,001-60,000	11 (18)	5 (17)	6 (19)	
	60,001-80,000	13 (21)	9 (30)	4 (13)	
	80,001-100,000	5 (8)	2 (7)	3 (10)	
	>100,000	11 (18)	5 (17)	6 (19)	
	No answer	12 (20)	4 (13)	8 (26)	
**Diagnosed with OA, n (%)**					>.99
	Yes	52 (85)	26 (87)	26 (84)	
	No, but met the “likely OA” criteria	9 (15)	4 (13)	5 (16)	
**In general, would you say your health is... n (%)**					.68
	Excellent	1 (2)	1 (3)	0	
	Very good	29 (48)	15 (50)	14 (45)	
	Good	24 (39)	10 (33)	14 (45)	
	Fair	7 (12)	4 (13)	3 (10)	
	Poor	0	0	0	
**Compared to one year ago, how would you rate your health in general now? n (%)**					.78
	Much better	4 (7)	2 (7)	2 (6)	
	Somewhat better	9 (15)	6 (20)	3 (10)	
	About the same	27 (44)	12 (40)	15 (48)	
	Somewhat worse	21 (34)	10 (33)	11 (36)	
	Much worse	0	0	0	
Number of comorbid conditions, median (IQR)		3.0 (2.0-4.0)	2.0 (1.0-4.0)	4.0 (2.0-5.0)	.06
Body mass index (kg/m^2^), mean (SD)		29.2 (5.1)	29.2 (5.5)	29.2 (4.8)	.95

^a^*P* values based on exact chi-square tests for categorical variables (nonmissing data) and two-sample *t* tests for continuous variables.

## Discussion

This proof-of-concept study showed that our intervention had a significant effect on time spent on physical activity (MVPA ≥3 METs) by people with knee OA compared to controls. The program also had a positive effect on step count, as well as activity of daily living and quality of life measured by the KOOS. Furthermore, the 2-month delay did not affect how participants responded to the program. This is noteworthy for the design of future RCTs in which the use of a “delay control” is considered. Our findings match those of previous studies that found individualized programs and self-management techniques could enhance physical activity adherence among people with chronic musculoskeletal conditions [16,17]. However, the findings should be viewed in the context that compared to the general population with knee OA, our participants were more active (baseline MVPA ≥3 METs for more than 60 minutes per day). A 2011 study in the United States using accelerometers found that more than 90% of people with knee OA failed to meet the physical activity guidelines of 150 minutes of MVPA per week [11].

Our positive results may be explained by two reasons. First, we employed a variety of behavior change techniques to promote physical activity. In a review of 13 consumer wearables, Lyons et al [42] found that Fitbit used behavior change techniques such as goal setting, feedback, self-monitoring, social support, social comparison, as well as providing instructions and rewards. Although many of these techniques are in line with recommendations from social cognitive theory [43], techniques such as action planning, problem solving, and behavioral practice/rehearsal are absent with the use of wearables alone [42]. In a 2015 systematic review, Lewis et al [44] reported that wearable-only interventions tend to produce only modest effects on improving physical activity behavior. Therefore, we included PT counselors with expertise in arthritis care and motivational interview skills to prompt participants to plan and practice their activities, and to identify potential barriers and solutions during the follow-up calls. Our findings also echo those of a recent RCT on a 12-week intervention involving an Internet-based physical activity program with the use of an accelerometer and remote coaching. Broekhuizen et al [45] reported that the intervention improved the emotional and mental health among community-dwelling older adults (mean age 65 years).

The second reason was related to the experience and training of our study PTs. Previous studies in health counseling have stressed the importance of the experience and skill of counselors in behavioral interventions [46,47]. In this study, all PTs had a clinical caseload primarily in arthritis, received the essential training on motivational interviewing, and were familiar with the counseling protocol. Taken together, these reasons might have contributed to the positive effects of the intervention.

However, we did not observe a significant effect in the mean time spent in sedentary activities. From the feasibility study, we learned that increasing physical activity and reducing sedentary behavior (eg, prolonged sitting) required distinct counseling approaches, and that practice would be required by the PTs to deliver the intervention [17]. Hence, all study PTs were provided opportunities to practice the counseling procedure with the investigators before data collection. We did not make major modification to the counseling protocol, and it was possible that some challenges persisted for participants to identify ways to break up their sitting time. It should be noted that sedentary behavior is linked to individuals’ habitual routines [48-51]. Although the general recommendation of MVPA can be accomplished in short daily episodes (eg, 30 minutes of brisk walking), reducing sedentary time requires people adjusting their habits throughout the day (eg, computer/mobile device use, television viewing). Therefore, different counseling strategies are likely required for the two behaviors.

In light of the findings, we suggest two modifications for the physical activity counseling program. First, the counseling conversation should begin by examining the individual’s habitual routines during a typical workday and a non-work/weekend day, and then focus on periods when prolonged sitting occurs. This would allow the conversation to center on identifying opportunities, challenges, and solutions to break up sitting time when it is feasible for the person. Second, a more flexible system would be needed for setting personal goals to reduce sedentary time. The current Fitbit-manufactured apps, both the Web-based and mobile versions, reward users if they take 250 steps or more (approximately 2-3 minutes of walking) in a given hour. Although the parameter meets the general recommendation of standing up and moving after 30 minutes of uninterrupted sitting, it does not allow the flexibility to individualize goals to break up sitting. We suggest that future Fitbit-compatible apps should include functions for users to set personalized goals on the frequency and duration to break up their sitting time during the day, and to receive feedback on their goal attainment. This would provide users with positive reinforcement to adopt the current recommendation of reducing extended periods of sitting.

This study has a few limitations. With the use of a delayed-control design in which the participants in the control arm received treatment after a 2-month delay, efficacy of the physical activity counseling intervention could only be assessed at 2 months. Hence, the long-term effect of the intervention remained unclear. Furthermore, our sample was relatively active; hence, the results may not be generalizable to people with knee OA who are more sedentary. The results also may not be generalizable to men because 82% of the participants were women. Finally, we have identified several shortcomings in the counseling program that may have limited its potential to affect the behavioral and health-related outcomes. Despite these limitations, this proof-of-concept study has demonstrated a significant effect of a multifaceted counseling intervention on improving physical activity participation. We have since applied these learnings to improve the next iteration of the program. Specifically, it now includes a new sedentary counseling strategy and a Fitbit-compatible Web app with enhanced functionality for setting goals and rewarding behaviors that break up prolonged sitting [52]. The modified program is currently being tested in a RCT involving people with rheumatoid arthritis and systematic lupus erythematosus (ClinicalTrial.gov identifier: NCT02554474) [53].

## Appendix

Multimedia Appendix 1 Participant outcomes and results of contrast analyses.

## Abbreviations

BMI: body mass index
ICC: intraclass correlation coefficient
KOOS: Knee Injury and Osteoarthritis Outcome Score
MET: metabolic equivalent
MVPA: moderate-to-vigorous physical activity
OA: osteoarthritis
PAR-Q: Physical Activity Readiness Questionnaire
PT: physical therapist
RCT: randomized controlled trial

## References

[1] Bombardier C, Hawker G, Mosher D (2011). The Impact of Arthritis in Canada: Today and Over the Next 30 Years.

[2] Brosseau L, MacLeay L, Robinson V, Wells G, Tugwell P (2003). Intensity of exercise for the treatment of osteoarthritis. Cochrane Database Syst Rev.

[3] Zhang W, Moskowitz RW, Nuki G, Abramson S, Altman RD, Arden N, Bierma-Zeinstra S, Brandt KD, Croft P, Doherty M, Dougados M, Hochberg M, Hunter DJ, Kwoh K, Lohmander LS, Tugwell P (2007). OARSI recommendations for the management of hip and knee osteoarthritis, part I: critical appraisal of existing treatment guidelines and systematic review of current research evidence. Osteoarthritis Cartilage.

[4] Roos E, Dahlberg L (2005). Positive effects of moderate exercise on glycosaminoglycan content in knee cartilage: a four-month, randomized, controlled trial in patients at risk of osteoarthritis. Arthritis Rheum.

[5] Munukka M, Waller B, Häkkinen Arja, Nieminen M, Lammentausta E, Kujala U, Paloneva J, Kautiainen H, Kiviranta I, Heinonen A (2017). Physical activity Is related with cartilage quality in women with knee osteoarthritis. Med Sci Sports Exerc.

[6] Skou S (2017). Roos EM: Good Life with osteoArthritis in Denmark (GLA:D)vidence-based education and supervised neuromuscular exercise delivered by certified physiotherapists nationwide. BMC Musculoskelet Disord.

[7] Davis AM, Kennedy D, Wong R, Robarts S, Skou SrT, McGlasson R, Li LC, Roos E (2018). Cross-cultural adaptation and implementation of Good Life with osteoarthritis in Denmark (GLA:D™): group education and exercise for hip and knee osteoarthritis is feasible in Canada. Osteoarthritis Cartilage.

[8] Zhang W, Moskowitz RW, Nuki G, Abramson S, Altman RD, Arden N, Bierma-Zeinstra S, Brandt KD, Croft P, Doherty M, Dougados M, Hochberg M, Hunter DJ, Kwoh K, Lohmander LS, Tugwell P (2008). OARSI recommendations for the management of hip and knee osteoarthritis, Part II: OARSI evidence-based, expert consensus guidelines. Osteoarthritis Cartilage.

[9] Haskell W, Lee I, Pate R, Powell K, Blair S, Franklin B, Macera C, Heath G, Thompson P, Bauman Adrian (2007). Physical activity and public health: updated recommendation for adults from the American College of Sports Medicine and the American Heart Association. Med Sci Sports Exerc.

[10] Wallis JA, Webster KE, Levinger P, Taylor NF (2013). What proportion of people with hip and knee osteoarthritis meet physical activity guidelines? A systematic review and meta-analysis. Osteoarthritis Cartilage.

[11] Dunlop DD, Song J, Semanik PA, Chang RW, Sharma L, Bathon JM, Eaton CB, Hochberg MC, Jackson RD, Kwoh CK, Mysiw WJ, Nevitt MC, Hootman JM (2011). Objective physical activity measurement in the osteoarthritis initiative: are guidelines being met?. Arthritis Rheum.

[12] Nelson AE, Allen KD, Golightly YM, Goode AP, Jordan JM (2014). A systematic review of recommendations and guidelines for the management of osteoarthritis: The chronic osteoarthritis management initiative of the U.S. bone and joint initiative. Semin Arthritis Rheum.

[13] Gyurcsik N, Brawley L, Spink K, Brittain D, Fuller D, Chad K (2009). Physical activity in women with arthritis: examining perceived barriers and self-regulatory efficacy to cope. Arthritis Rheum.

[14] Der Ananian C, Wilcox S, Saunders R, Watkins K, Evans A (2006). Factors that influence exercise among adults with arthritis in three activity levels. Prev Chronic Dis.

[15] Henchoz Y, Zufferey P, So A (2013). Stages of change, barriers, benefits, and preferences for exercise in RA patients: a cross-sectional study. Scand J Rheumatol.

[16] Jordan J, Holden M, Mason EJ, Foster NE (2010). Interventions to improve adherence to exercise for chronic musculoskeletal pain in adults. Cochrane Database Syst Rev.

[17] Li L, Sayre E, Xie H, Clayton C, Feehan LM (2017). A community-based physical activity counselling program for people with knee osteoarthritis: feasibility and preliminary efficacy of the track-OA study. JMIR Mhealth Uhealth.

[18] Marra C, Cibere J, Tsuyuki R, Soon J, Esdaile J, Gastonguay L, Oteng B, Embley P, Colley L, Enenajor G, Kok R (2007). Improving osteoarthritis detection in the community: pharmacist identification of new, diagnostically confirmed osteoarthritis. Arthritis Rheum.

[19] Altman R, Asch E, Bloch D, Bole G, Borenstein D, Brandt K, Christy W, Cooke T, Greenwald R, Hochberg M (1986). Development of criteria for the classification and reporting of osteoarthritis. Classification of osteoarthritis of the knee. Diagnostic and Therapeutic Criteria Committee of the American Rheumatism Association. Arthritis Rheum.

[20] Thomas S, Reading J, Shephard R J (1992). Revision of the Physical Activity Readiness Questionnaire (PAR-Q). Can J Sport Sci.

[21] Rollnick S, Miller W, Butler CC (2009). Motivational Interviewing in Health Care: Helping Patients Change Behavior.

[22] Miller W, Rollnick S (2012). Motivational Interviewing: Helping People Change. 3rd ed.

[23] Richards J, Hillsdon M, Thorogood M, Foster C (2013). Face-to-face interventions for promoting physical activity. Cochrane Database Syst Rev.

[24] Gutnick D, Reims K, Davis C, Gainforth H, Jay M, Cole S (2014). Brief action planning to facilitate behavior change and support patient self-management. J Clin Outcomes Manag.

[25] Tierney M, Fraser A, Purtill H, Kennedy Norelee (2013). Study to determine the criterion validity of the SenseWear Armband as a measure of physical activity in people with rheumatoid arthritis. Arthritis Care Res (Hoboken).

[26] Holsgaard-Larsen A, Roos EM (2012). Objectively measured physical activity in patients with end stage knee or hip osteoarthritis. Eur J Phys Rehabil Med.

[27] Semanik P, Lee J, Manheim L, Dipietro L, Dunlop D, Chang RW (2011). Relationship between accelerometer-based measures of physical activity and the Yale Physical Activity Survey in adults with arthritis. Arthritis Care Res (Hoboken).

[28] Almeida G, Wasko M, Jeong K, Moore C, Piva Sara R (2011). Physical activity measured by the SenseWear Armband in women with rheumatoid arthritis. Phys Ther.

[29] Ainsworth B, Haskell EW Compendium of Physical Activities Tracking Guide.

[30] Tremblay MS, Aubert S, Barnes JD, Saunders TJ, Carson V, Latimer-Cheung AE, Chastin SF, Altenburg TM, Chinapaw MJ, SBRN Terminology Consensus Project Participants (2017). Sedentary Behavior Research Network (SBRN) - Terminology Consensus Project process and outcome. Int J Behav Nutr Phys Act.

[31] Roos E, Roos P, Lohmander L, Ekdahl C, Beynnon BD (1998). Knee Injury and Osteoarthritis Outcome Score (KOOS)--development of a self-administered outcome measure. J Orthop Sports Phys Ther.

[32] Roos E, Roos P, Ekdahl C, Lohmander LS (1998). Knee injury and Osteoarthritis Outcome Score (KOOS)--validation of a Swedish version. Scand J Med Sci Sports.

[33] Petkov J, Harvey P, Battersby M (2010). The internal consistency and construct validity of the partners in health scale: validation of a patient rated chronic condition self-management measure. Qual Life Res.

[34] Feehan L, Goldsmith CH, Leung AF, Li LC (2016). SenseWearMini and Actigraph GT3X accelerometer classification of observed sedentary and light-intensity physical activities in a laboratory setting. Physiother Can.

[35] Owen N (2012). Sedentary behavior: understanding and influencing adults' prolonged sitting time. Prev Med.

[36] Dunstan D, Kingwell B, Larsen R, Healy G, Cerin E, Hamilton M, Shaw J, Bertovic D, Zimmet P, Salmon J, Owen N (2012). Breaking up prolonged sitting reduces postprandial glucose and insulin responses. Diabetes Care.

[37] Latouche C, Jowett J, Carey A, Bertovic D, Owen N, Dunstan D, Kingwell BA (2013). Effects of breaking up prolonged sitting on skeletal muscle gene expression. J Appl Physiol (1985).

[38] Howard B, Fraser S, Sethi P, Cerin E, Hamilton M, Owen N, Dunstan D, Kingwell BA (2013). Impact on hemostatic parameters of interrupting sitting with intermittent activity. Med Sci Sports Exerc.

[39] Ory M, Resnick B, Jordan P, Coday M, Riebe D, Ewing GC, Pruitt L, Bazzarre T (2005). Screening, safety, and adverse events in physical activity interventions: collaborative experiences from the behavior change consortium. Ann Behav Med.

[40] Schoenfeld D (1980). Statistical considerations for pilot studies. Int J Radiat Oncol Biol Phys.

[41] Carter R, Woolson RF (2004). Statistical design considerations for pilot studies transitioning therapies from the bench to the bedside. J Transl Med.

[42] Lyons J, Lewis H, Mayrsohn G, Rowland JL (2014). Behavior change techniques implemented in electronic lifestyle activity monitors: a systematic content analysis. J Med Internet Res.

[43] Task Force on Community Preventive Services (2002). Recommendations to increase physical activity in communities. Am J Prev Med.

[44] Lewis Z, Lyons E, Jarvis J, Baillargeon J (2015). Using an electronic activity monitor system as an intervention modality: A systematic review. BMC Public Health.

[45] Broekhuizen K, de Gelder J, Wijsman C, Wijsman L, Westendorp R, Verhagen E, Slagboom P, de Craen A, van Mechelen W, van Heemst D, van der Ouderaa F, Mooijaart SP (2016). An Internet-based physical activity intervention to improve quality of life of inactive older adults: a randomized controlled trial. J Med Internet Res.

[46] Tulloch H, Fortier M, Hogg W (2006). Physical activity counseling in primary care: who has and who should be counseling?. Patient Educ Couns.

[47] Hutchison A, Breckon J, Johnston LH (2009). Physical activity behavior change interventions based on the transtheoretical model: a systematic review. Health Educ Behav.

[48] O'Donoghue G, Perchoux C, Mensah K, Lakerveld J, van der Ploeg H, Bernaards C, Chastin S, Simon C, O'Gorman D, Nazare J, DEDIPAC Consortium (2016). A systematic review of correlates of sedentary behaviour in adults aged 18-65 years: a socio-ecological approach. BMC Public Health.

[49] Rutten G, Savelberg H, Biddle S, Kremers SP (2013). Interrupting long periods of sitting: good STUFF. Int J Behav Nutr Phys Act.

[50] Gardner B, Smith L, Lorencatto F, Hamer M, Biddle SJ (2016). How to reduce sitting time? A review of behaviour change strategies used in sedentary behaviour reduction interventions among adults. Health Psychol Rev.

[51] Gardner B, Lally P, Wardle J (2012). Making health habitual: the psychology of 'habit-formation' and general practice. Br J Gen Pract.

[52] Gupta A, Tong X, Shaw C, Li L, Feehan L, Stephanidis C (2017). HCI International 19th International Conference, Proceedings, Part II.

[53] Li LC, Feehan LM, Shaw C, Xie H, Sayre EC, Aviña-Zubeita A, Grewal N, Townsend AF, Gromala D, Noonan G, Backman Cl (2017). A technology-enabled counselling program versus a delayed treatment control to support physical activity participation in people with inflammatory arthritis: study protocol for the OPAM-IA randomized controlled trial. BMC Rheumatol.

