# Supplementary material for: Efficacy of a Community-Based Technology-Enabled Physical Activity Counseling Program for People With Knee Osteoarthritis: Proof-of-Concept Study
Source: J Med Internet Res. 2018 Apr 30;20(4):e159. doi: 10.2196/jmir.8514 (PMC5952118; doi:10.2196/jmir.8514)
Supplement: Multimedia Appendix 1 [file jmir_v20i4e159_app1.pdf]

Participant outcomes and results of contrast analyses

	**Immediate Group (n = 30)**				**Delay Group (n = 31)**				**Group effect Immediate Group vs. Delayed Group Coefficient (95% CI) §**				
	**Baseline**	**2 months**	**4 months**	**6 months**	**Baseline**	**2 months**	**4 months**	**6 months**	**Cont. 1**	**Cont. 2**	**Cont. 3**	**Cont. 4**	**Cont. 5**
**Average daily bouted MVPA†: 3+ METs [mins]**	62.1 (54.6)	75.5 (54.3)	62.6 (56.3)	65.6 (48.5)	65.3 (77.4)	50.0 (46.8)	60.1 (76.8)	70.7 (71.9)	26.6* (4.0, 49.1)	25.5 (-2.4, 53.5)	26.0* (3.1, 49.0)	1.0 (-20.7, 22.7)	0.2 (-10.6, 11.0)
**Average daily bouted MVPA†: 4+ METs [mins]**	24.0 (30.9)	29.2 (32.9)	25.0 (35.1)	20.3 (32.9)	16.8 (30.7)	10.8 (19.2)	18.9 (34.3)	18.3 (28.7)	8.2 (-2.2, 18.5)	12.2 (-2.3, 26.8)	10.2 (-0.9, 21.3)	-4.1 (-16.2, 8.1)	-1.7 (-7.2, 3.8)
**Average daily steps**	7,069.2 (3,375.3)	8,217.4 (3,095.5)	8,132.5 (3,420.7)	8,215.1 (3,725.6)	7,556.6 (5,054.1)	6,713.6 (3,354.3)	7,631.9 (4,054.3)	7,573.6 (4,477.1)	1,699.2* (349.0, 3,049.4)	1,601.8* (38.7, 3164.9)	1,650.5* (332.3, 2,968.7)	97.4 (-1,160.5, 1,355.3)	37.6 (-532.1, 607.3)
**Average daily bouted Sedentary time‡ [mins]**	464.1 (137.7)	437.6 (133.9)	505.9 (167.2)	435.8 (138.8)	497.4 (200.7)	503.0 (160.7)	508.9 (189.0)	496.9 (179.5)	-20.9 (-63.4, 21.5)	6.4 (-49.2, 61.9)	-7.3 (-50.4, 35.8)	-27.3 (-75.7, 21.1)	24.5 (-3.8, 52.9)
**KOOS (0-100; higher = better)**													
**Symptoms**	59.8 (16.1)	62.6 (15.6)	62.4 (14.7)	62.1 (15.3)	62.9 (17.2)	61.7 (14.5)	63.4 (16.7)	61.4 (19.8)	4.5 (-1.7, 10.7)	3.8 (-4.0, 11.5)	4.1 (-2.2, 10.4)	0.7 (-5.5, 7.0)	-0.6 (-3.7, 2.4)
**Pain**	66.2 (17.5)	70.9 (17.0)	67.5 (15.5)	68.6 (17.5)	65.1 (17.9)	64.8 (14.6)	66.3 (15.2)	66.2 (16.4)	4.4 (-2.7, 11.6)	1.7 (-6.6, 10.0)	3.1 (-3.8, 10.0)	2.8 (-4.1, 9.6)	-1.8 (-5.1, 1.4)
**ADL**	71.8 (17.5)	76.0 (16.1)	76.6 (17.5)	75.1 (15.9)	74.1 (17.6)	71.0 (16.5)	75.2 (17.2)	73.9 (15.8)	6.9* (0.1, 13.7)	7.5 (-0.1, 15.1)	7.2* (0.8, 13.6)	-0.6 (-7.1, 5.8)	-0.6 (-3.4, 2.2)
**Sports & recreation**	47.3 (26.6)	49.3 (24.9)	50.0 (25.6)	50.4 (26.3)	52.7 (27.7)	47.0 (23.3)	48.9 (27.7)	49.8 (29.2)	6.9 (-3.7, 17.5)	8.1 (-5.3, 21.4)	7.5 (-3.2, 18.2)	-1.2 (-12.5, 10.1)	0.9 (-5.9, 7.6)
**Qol**	41.0 (19.8)	47.2 (18.9)	45.4 (17.6)	44.9 (17.9)	44.6 (16.3)	42.4 (16.8)	47.5 (13.6)	48.1 (19.1)	7.4* (0.0, 14.7)	7.2 (-1.3, 15.8)	7.3* (0.1, 14.6)	0.1 (-6.6, 6.9)	-0.8 (-4.1, 2.5)
**Partners in Health (0-96; lower = better)**	18.4 (12.6)	14.9 (11.4)	14.3 (10.1)	12.9 (9.2)	20.5 (12.8)	18.3 (13.7)	14.9 (9.6)	13.1 (9.2)	-1.6 (-6.3, 3.2)	-1.0 (-6.5, 4.5)	-1.3 (-6.0, 3.4)	-0.5 (-4.9, 3.9)	-1.0 (-3.0, 1.0)

Standard deviations are in brackets

† MVPA (moderate/vigorous physical activity) was performed in bouts ≥ 10 minutes

‡ Sedentary behaviours were performed in bouts > 20 minutes

§ Contrast analyses:

- Contrast 1: Immediate Group T1 – T0 vs. Delayed Group T1 – T0
- Contrast 2: Delayed Group T2 – T1 vs. Delayed Group T1 – T0

- Contrast 3: Average of Contrast 1 and Contrast 2
- Contrast 4 : Immediate Group T1 – T0 vs. Delayed Group T2 – T1
- Contrast 5 : Average of Immediate Group T2 – T1 and Delayed Group T3 – T2

* $p < 0.05$
